# Computational models of neuron-astrocyte interaction in epilepsy

**DOI:** 10.3389/fncom.2012.00058

**Published:** 2012-08-13

**Authors:** Vladislav Volman, Maxim Bazhenov, Terrence J. Sejnowski

**Affiliations:** ^1^Computational Neurobiology Laboratory, Howard Hughes Medical Institute, The Salk Institute for Biological StudiesLa Jolla, CA, USA; ^2^Center for Theoretical Biological Physics, University of California at San DiegoLa Jolla, CA, USA; ^3^L-3 Applied Technologies/Simulation, Engineering, and TestingSan Diego, CA, USA; ^4^Department of Cell Biology and Neuroscience, University of California at RiversideRiverside, CA, USA; ^5^Division of Biological Sciences, University of California at San DiegoLa Jolla, CA, USA

**Keywords:** astrocytes, regulatory signaling, epileptogenesis, trauma, inflammation

## Abstract

Astrocytes actively shape the dynamics of neurons and neuronal ensembles by affecting several aspects critical to neuronal function, such as regulating synaptic plasticity, modulating neuronal excitability, and maintaining extracellular ion balance. These pathways for astrocyte-neuron interaction can also enhance the information-processing capabilities of brains, but in other circumstances may lead the brain on the road to pathological ruin. In this article, we review the existing computational models of astrocytic involvement in epileptogenesis, focusing on their relevance to existing physiological data.

## Introduction

There has been an increasing effort to understand the etiology of brain disorders using computational models of the neural circuits that can predict the impact of changes in intrinsic neuronal properties and interneuronal coupling on the dynamics of neuronal networks. The ability of biophysically detailed models to address working hypotheses for brain disorders is especially important given the methodological and ethical concerns associated with the experimental studies on humans.

Models based on the biophysical underpinnings of brain pathologies, focusing on neurons, and neuronal networks, are useful in determining how different intrinsic and network properties can result in pathological activity. For example, in models of cortical networks, an imbalance in the ratio of fast excitation and inhibition can lead to epileptic seizures; however, this approach is primarily based on the relatively fast neuronal dynamics and thus cannot explain how the slow (>days) transition from normal to pathological state occurs. In contrast, some brain disorders (e.g., epilepsy and schizophrenia to provide few examples) may result from aberrations in slow, homeostatic, mechanisms that modulate the function of single neurons and properties of synaptic plasticity/connectivity. Thus, though useful, models of fast neuronal dynamics provide only limited insight into slowly developing pathological states of the brain, and a paradigm shift is needed for computational approaches to succeed in revealing the mechanisms underlying brain pathologies.

Neurons are critical for information processing in the human brain, but they comprise less than half of brain mass, which also includes glial cells. Glial cells are further subdivided into microglia, oligodendrocytes, Schwann cell, and astrocytes. Astrocytes are the most numerous glial cell type and account for up to one third of brain mass (Kandel, 1991). Because of their important role in glucose metabolism and maintenance of extracellular ion homeostasis, astrocytes have been viewed as “support cells,” secondary to neurons and having little to do with brain activity *per se*. However, growing experimental evidence [reviewed in Seifert et al. (2006); Giaume et al. (2010); Halassa and Haydon (2010)] points to the possibility that glial cells (and astrocytes in particular) are actively involved in the modulation of synaptic transmission and neural excitability. This has led researchers to propose that astrocytes can dynamically redefine the functional architecture of neuronal networks (Nedergaard et al., 2003).

Unlike the fast millisecond time scale for action potential and fast chemical transmission, astrocytes are regulated mainly through calcium signaling on a much slower time scale of seconds to minutes, consistent with the slow time scale associated with homeostatic regulation of neuronal activity. Furthermore, astrocytes respond to chronic changes in neuronal activity (such as those incurred by trauma) by releasing various pro-inflammatory molecules that offset the perturbation. Thus, astrocyte-neuron signaling appears to be a plausible setting to explain at least part of the etiology of brain diseases linked to the aberrations in homeostatic regulation.

This review attempts to survey existing computational models of astrocytic involvement in disorders of the nervous system, with particular emphasis on models of epileptogenesis. The dynamical signature of epilepsy is hypersynchronization of collective neuronal activity, suggesting a breakdown in homeostasis. We also provide a perspective on what is needed to better understand how these non-neuronal cells contribute to brain pathologies.

## Modulation of synaptic transmission

Although hyperexcitability of individual neurons can significantly contribute to epileptogenesis, epilepsy is widely considered to be a “network disease” driven by aberrant synaptic interaction between neurons (McCormick and Contreras, 2001). Synaptic plasticity (in particular short-term synaptic depression and facilitation) can critically mold the functional strength of synaptic coupling; thus, it is possible that specific properties of synaptic transmitter release could predispose a network to seizures.

**Figure 1:**
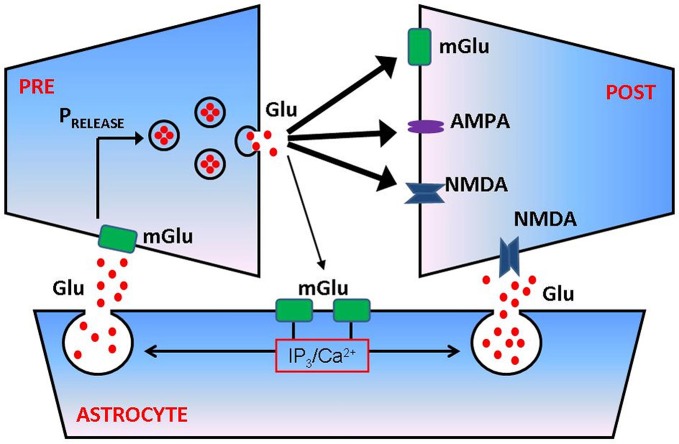
**Astrocytes regulate presynaptic transmission and postsynaptic excitability at glutamatergic synapse.** Following successful vesicular fusion, a fraction of released glutamate can spill over and reach metabotropic glutamate (mGlu) receptors on the membrane of adjacent astrocyte. Glutamate activation of astrocytic mGlu receptors activates a cascade of biochemical events that culminates in elevation of inositol trisphosphate (IP3) and increased intra-astrocytic free calcium concentration. Calcium causes a release of astrocytic glutamate (a process termed gliotransmission) which can bind mGlu receptors on presynaptic membrane and/or NMDA receptors on postsynaptic side, thus modulating the probability of synaptic transmitter release (P_RELEASE_) and/or postsynaptic excitability. For more quantitative details regarding the process of astrocyte modulation of synaptic transmission, see De Pitta et al. (2011).

Astrocytes are ideally positioned to modulate synaptic contributions to epileptogenesis. Experimental data [reviewed in Araque et al. (1999)] shows that astrocytic processes are often apposed to synaptic junctions, giving rise to the notion of “tripartite synapse” consisting of presynaptic bouton, the astrocyte, and the postsynaptic density. Stimulation of synaptic boutons and subsequent release of neurotransmitter elicits complex calcium responses in nearby astrocytes, showing that these glial cells can “eavesdrop” on synaptic activity [reviewed in Haydon (2001)]. Elevation of astrocytic calcium culminates in the regulated release of molecules such as glutamate and/or ATP which, by diffusing in the extracellular space (ECS) and binding to dedicated receptors, can modulate the synaptic transmission (Araque et al., 1998) and the excitability of adjacent neurons (Parpura and Haydon, 2000; Reyes and Parpura, 2008) (Figure 1). It was shown recently that NMDA-R mediated astrocytic modulation of postsynaptic neuronal excitability can promote epileptogenesis in neuronal networks (Gomez-Gonzalo et al., 2010). This suggests the role for tripartite synapses not only in epilepsy, but perhaps also in other diseases of the nervous system that are based on aberrant synaptic communication (Halassa et al., 2007).

The first, to our knowledge, computational model that explored the potential involvement of astrocytes in synaptic mechanisms of epileptogenesis incorporated the basic notion of astrocytic “eavesdropping” on neuronal activity and assumed that the sole effect of neuronal activity-dependent astrocytic calcium elevations was to promote neuronal depolarization (Nadkarni and Jung, 2003; Appendix). This study concluded that astrocytes promote epileptogenesis through positive feedback. Similar conclusions were reached in another modeling study that showed that glutamate released from astrocytes could be responsible for paroxysmal depolarization shifts, often associated with epileptic activity (Silchenko and Tass, 2008). However, gliotransmitter released from astrocytes can either up- or down-regulate the release of glutamate from synaptic boutons (Araque et al., 1998; Zhang et al., 2003), and computational modeling studies suggested that such negative feedback modulation of synaptic transmission could account for the regulation of spontaneous paroxysmal activity in neural cultures (Volman et al., 2007). Thus, in the context of the tripartite synapse, astrocytic signaling could have either positive or negative impact on neuronal activity, or perhaps a combination of both. Revealing the mechanisms behind this diversity of modulating effects could help in understanding the conditions for seizure suppression or promotion by astrocytes.

Synaptic neurotransmitter release is a calcium-driven process; thus, a change in presynaptic calcium levels through glial modulation (for example by astrocytic activation of presynaptic receptor channels) could affect synaptic transmission. A computational modeling of a tripartite synapse supported the possibility that astrocytes may optimize the synaptic transmission of information by affecting the levels of presynaptic calcium (Nadkarni et al., 2008). In another recent modeling study (De Pitta et al., 2011), astrocytes either depressed or facilitated synaptic transmission, which suggested that the ultimate effect of astrocytes on transmitter release probability was determined by the interplay between the different mGlu-Rs (located presynaptically) and the baseline synaptic vesicle release probability. Incorporating these pathways, the model was successful at explaining several contradictory experimental observations regarding the role of astrocytes in the modulation of synaptic transmission.

## Regulation of extracellular ion and water levels

**Figure 2:**
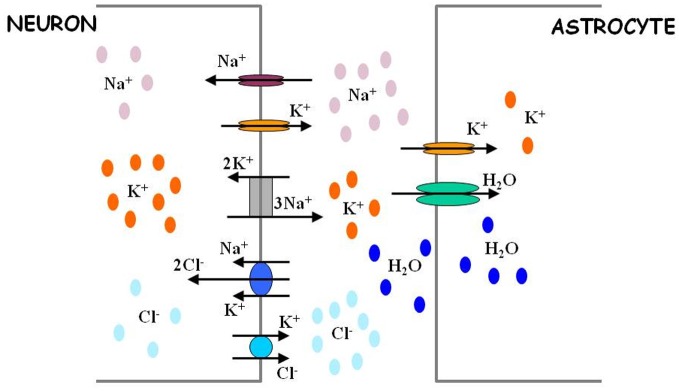
**Astrocytes regulate extracellular ion concentrations and water level.** Neuronal excitability is defined in part by activity-dependent Nernst potentials of sodium (Na^+^), potassium (K^+^), and chloride (Cl^−^) ions. The intracellular and extracellular concentrations of these ions are critically determined by the action of different ion transport mechanisms (sodium potassium pump and chloride co-transporters). Astrocytes possess potassium channels and thus are in position to regulate the levels of extracellular potassium. Through this regulation, astrocytes can also indirectly affect the levels of intracellular chloride ions in neurons. The efficacy of extracellular potassium regulation by astrocytes may be achieved by water-dependent regulation of extracellular space, as explained in Text. For more quantitative details regarding the process of ion level regulation by astrocytes, follow Ostby et al. (2009).

Besides eavesdropping on synaptic activity, astrocytes also possess the means to “estimate” the level of neuronal activity in their proximity. The shape of a neuronal action potential is governed by ionic gradients across the membrane, but, in turn, the action potential itself causes changes in local ion concentrations (Figure 2). The principal effect is a spike-activity-dependent accumulation of potassium ions in the ECS. Astrocytes maintain potassium homeostasis by employing inward rectifying potassium channels to take up the excess K^+^ that accumulates following action potential activity and redistributing K^+^ ions across space compartments. Thus, astrocytes can monitor the level of neuronal activity through changes in extracellular potassium concentration.

Unlike astrocytic sensing of synaptic activity (which can in principle play a regulatory role in the flow of neural information, as mentioned below), astrocytic regulation of extracellular ion concentration is mostly associated with neuropathology. Physiological experiments and theoretical arguments (Frankenhaeuser and Hodgkin, 1956) show that elevated extracellular potassium can significantly increase the reversal potential of potassium channel conductance, thus rendering potassium currents depolarizing. Cumulatively, these observations led to the “high K^+^” theory of epileptogenesis (Fisher et al., 1976; Lothman and Somjen, 1976), according to which activity-dependent elevation of extracellular potassium acts as a positive feedback mechanism promoting dynamical instability that culminates in seizure generation (Janigro et al., 1997) [reviewed in Frohlich et al. (2008b)]. Because astrocytes are involved in setting potassium levels (Janigro et al., 1997), it is imperative to understand how astrocytes contribute to “high K^+^” epileptogenesis.

Early computational models that explored the role of high K^+^ in epileptogenesis (Kager et al., 2000; Bazhenov et al., 2004; Frohlich et al., 2006, 2010) focused on the dynamics of single neurons and neuronal networks and made simplistic assumptions regarding the astrocytic mechanisms for potassium regulation (Appendix). More recent models have incorporated more diverse mechanisms of ion movement in single neurons (Cressman et al., 2009; Somjen et al., 2008; Krishnan and Bazhenov, 2011) and ion exchange between single neurons and astrocytes (Somjen et al., 2008; Oyehaug et al., 2011) (Figure 2). The interrelated dynamics of sodium and potassium currents during epileptogenesis were examined in a network model that incorporated both neuronal and glial cells (Ullah et al., 2009). Although, these studies highlighted different aspects of ion concentration regulation by astrocytes (pumps, transporters, etc.), they all converged to the same conclusion—the failure of astrocytes to maintain proper ionic micro-environment in neuronal surrounding can be critical for initiating epileptogenesis through the “high K^+^” mechanism.

Several lines of evidence suggest that astrocytes could also affect potassium-mediated epileptogenesis through regulation of extracellular water levels (Simard and Nedergaard, 2004). Water movement in the brain is likely to involve aquaporin 4 (AQP4) channels, which are widely expressed in astrocytes (Nagelhus et al., 2004). Interestingly, AQP4 channels in astrocytes are co-localized with potassium Kir4.1 channels (Nagelhus et al., 2004). Furthermore, potassium clearance was significantly compromised in AQP4 knockout mice (Binder et al., 2006). In these AQP4^−/−^ mice, the threshold for seizure generation was higher, but seizure duration was longer compared to controls (Amiry-Moghaddam et al., 2003; Binder et al., 2006). This suggests that astrocytic regulation of extracellular water could contribute to epileptogenesis during intense neuronal activity through the depolarization of astrocytes by the accumulation of extracellular K^+^ ions. This depolarization would activate the electrogenic sodium bicarbonate cotransporter, favoring the uptake of these ions by the astrocyte. The uptake of sodium and bicarbonate ions by astrocytes would establish an osmolarity gradient (higher intracellular osmolarity) and thus would drive the water into glial cells through AQP4 channels, eventually contributing to astrocytic swelling and shrinkage of ECS. Activity-dependent shrinkage of ECS implies a smaller distribution volume for extracellular potassium, thus lowering the seizure threshold. The implications of activity-dependent, astrocyte-mediated shrinkage of ECS were recently explored in a computational model that incorporated a variety of astrocytic ion exchange mechanisms and water regulation (Ostby et al., 2009).

Epileptogenesis has also been shown to be facilitated by the intracellular accumulation of chloride in neurons (Dzhala et al., 2005). The electrochemical gradient of chloride defines the reversal potential of gamma-aminobutyric acid (GABA) receptor-mediated chloride currents, and thus determines the extent to which GABAergic synaptic signaling (normally assumed to have hyperpolarizing seizure-suppressing influence) is inhibitory. During physiologically normal activity, the intracellular chloride concentration is much lower than its extracellular levels, keeping E_Cl_ low and thus ensuring hyperpolarizing effect of GABAergic synapse. The high levels of activity that occur during seizures results in intracellular chloride accumulation and a higher GABA reversal potential, which can potentially lead to depolarizing GABA currents. In early development, intracellular chloride concentration is so high that GABA has a depolarizing effect on the neuronal membrane potential (Ben-Ari and Holmes, 2005). Increase of intracellular chloride concentration can also directly affect the resting potential and excitability of neurons. Using model incorporating Na^+^, K^+^, and Cl^−^ concentration dynamics, it was shown that an increase of intracellular chloride concentration extends seizure duration making possible long-lasting epileptic activity characterized by multiple transitions between tonic and clonic states (Krishnan and Bazhenov, 2011).

The impact of chloride on epileptogenesis primarily thus depends on the homeostasis of *intracellular* chloride concentration. Because astrocytes cannot directly access ion levels in neurons, astrocytes are not expected to be involved in chloride mechanisms in epileptogenesis. However, chloride homeostasis in neurons is affected by the opposing action of cation-chloride co-transporters, NKCC1 (importing two ions of chloride along with one ion of sodium and one ion of potassium) and KCC2 (exporting one ion of chloride along with one ion of potassium) (Payne et al., 2003) (Figure 2). The relative expression of these different chloride co-transporters directly affects seizure generation (Glykys et al., 2009; Dzhala et al., 2010). In particular, KCC2 co-transporter can mediate an effective coupling between extracellular potassium concentration and intracellular chloride concentration: a higher extracellular potassium level leads to higher intracellular chloride concentration (Payne et al., 2003). Thus, a dysfunction in extracellular potassium uptake by astrocytes could indirectly facilitate the destabilizing effect of intracellular chloride on a neuron's spiking activity.

## Astrocytes in post-traumatic epilepsy

Traumatic brain injury (TBI) (e.g., as a result of penetrative wound) can increase the predisposition to epileptic seizures after a latent period following a traumatic event (Annegers et al., 1998). Although there is a clear causal link between TBI events and the later emergence of epileptic seizures, the etiology of “post-traumatic epilepsy” remains elusive (Agrawal et al., 2006).

A possible immediate outcome of TBI is the massive death of neuronal cells and damage to synaptic connectivity between neurons, which can create areas with chronic neuronal and synaptic inactivity. Evidence from *in vitro* studies suggests that chronic inactivity can modify several parameters of neuronal circuitry (e.g., synaptic connectivity, synaptic conductances, local neuronal morphology, intrinsic neuronal excitability) to compensate for the loss of activity incurred by trauma. As a general rule, chronic inactivity promotes the upregulation of depolarizing influences and downregulation of hyperpolarizing influences, while over-excitation leads to downregulation of depolarizing influences and upregulation of hyperpolarizing influences. This suggests that homeostatic regulatory pathways may be activated after traumatic event to offset the perturbation in electrical activity (Turrigiano, 1999). Regional homeostatic adjustments of neuronal excitability and synaptic transmission could contribute to breaching the excitation-inhibition balance (Fritschy, 2008), thus promoting seizure generation in traumatized networks (Timofeev et al., 2010). Understanding the mechanisms of trauma-triggered homeostatic plasticity could therefore generate insights into the etiology of post-traumatic epilepsy.

Several computational modeling studies have addressed the role of homeostatic plasticity in post-traumatic epilepsy. In one model of trauma that included cell death, post-traumatic axonal sprouting led to the emergence of seizure-like activity in an otherwise non-seizing network (Santhakumar et al., 2004; Schneider-Mizell et al., 2010). This is consistent with earlier theories showing that network topology of synaptic connectivity can critically determine its seizing propensity (Netoff et al., 2004). In deafferentation models of cortical trauma (mimicking loss of excitation and chronic reduction of neuronal activity), homeostatic scaling of synaptic conductances could restore the “normal” firing rates, but this came at the expense of paroxysmal bursting (Houweling et al., 2005; Frohlich et al., 2008a; Appendix). Interestingly, the rate of paroxysmal activity depended on both intrinsic neuronal parameters (Houweling et al., 2005) and the spatial pattern of trauma (Volman et al., 2011b,c). Although these studies addressed different aspects of post-traumatic reorganization, all supported the notion that trauma-triggered homeostatic regulation can contribute to post-traumatic epileptogenesis.

**Figure 3:**
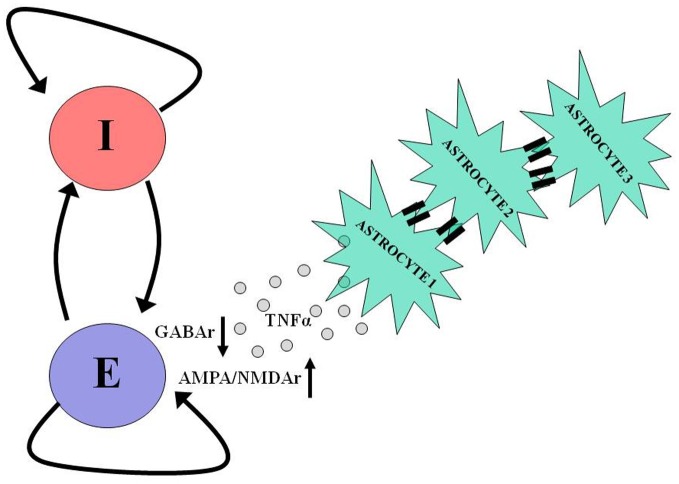
**Astrocytes take part in neuronal homeostatic plasticity.** In response to prolonged neuronal and synaptic inactivity, as occurs following trauma, astrocytes release tumor necrosis factor alpha (TNFα), which diffuses and binds to its dedicated receptors on pyramidal neurons causing a reduction in the number of postsynaptic GABA receptors and an increase in the number of postsynaptic AMPA and NMDA receptors. Such homeostatic modulation aims to offset the decrease in neuronal activity by shifting the excitation-inhibition balance in favor of excitation. The participation of astrocytes in homeostatic plasticity can be further facilitated by slow (10s of seconds) intercellular communication via gap junctions between these cells. For more details regarding the role of astrocytes in homeostatic plasticity and intercellular communication, see Pannasch et al. (2011).

Recent *in vitro* studies show that glial cells are critically involved in the homeostatic scaling of synaptic conductances that follows prolonged synaptic inactivity (Beattie et al., 2002; Stellwagen and Malenka, 2006; Steinmetz and Turrigiano, 2010). Following chronic synaptic inactivity, astrocytes release tumor necrosis factor alpha (TNFα) which diffuses and acts on postsynaptic neurons to scale up the number of AMPA/NMDA receptors and scale down the number of GABAa receptors (Figure 3). Thus, signaling by TNFα could represent a homeostatic regulatory mechanism compensating for a chronic reduction in neural excitability and could contribute to post-traumatic epileptogenesis. An observation in support of this hypothesis is that TNFα is released by glial cells early (1–2 h) after trauma, as a part of neuroinflammatory cascade. Computational models constructed with variable “glia” to explore this hypothesis concluded that glial release and diffusion of TNFα could affect post-traumatic paroxysmal activity (Savin et al., 2009).

## The next frontier

### Modeling the effects of cell morphology

In the first-generation computational models of astrocyte-neuron interaction, astrocytes and neurons were usually assumed to be point entities, neglecting completely their intracellular organization and dynamics. This simplifying assumption allowed the modulation of basic neural functions by astrocytes to be explored. However, the spatial aspects of cellular organization may also be important to understanding how glial cells affect brain pathologies. For example, the spatial co-localization of potassium and water transport mechanisms in astrocytes is involved in the regulation of traumatic edema and consequent post-traumatic epilepsy, as mentioned above.

Another elegant example of the importance of spatial organization is derived from energy considerations (Hertz et al., 2007). The cell body of a typical astrocyte accounts for only ~2% of its total volume, with larger, branching organelle-containing processes constituting ~60% (Hertz et al., 2007). The remaining ~40% are taken by tiny (~3 microns length by ~0.2 microns width) filopodia and lamellipodia which cannot accommodate energy-efficient organelles such as mitochondria. This implies that the terminal thin processes (which regulate synaptic function) and thicker organelle-containing branches use different energy strategies for their function (Hertz et al., 2007). This could have important implications for the ability of astrocytes to modulate synaptic function.

In experimental models of epilepsy, astrocytes undergo significant rapid morphological remodeling following the traumatic event (Oberheim et al., 2008), a process known as reactive gliosis (Sofroniew, 2009). Specifically, astrocytes that are located relatively close (200 microns) to the boundary between intact and injured cortical regions lose their trademark star shape and elongate in the direction perpendicular to the trauma boundary (Oberheim et al., 2008). The implications of this remodeling for network function remain largely unknown, but it was recently proposed that trauma-induced morphological reorganization of astrocytes could reduce the incidence of paroxysmal activity by providing better functional segregation of synaptic input from intact vs. injured neurons (Volman et al., 2011d). Functional segregation of synaptic input could help localize the release of TNFα to the regions of synaptic inactivity and thus prevent pathological over-excitation of relatively intact areas.

Modeling the microphysiology of astrocyte-neuron interactions is a challenging problem without knowing the distribution of different proteins on the surfaces of astrocytes and the movement of ions across membranes. One promising approach is based on Monte Carlo simulations of cellular microphysiology, which can explore the impact of different astrocytic morphologies (Nadkarni et al., 2010).

### Neuro-glial networks

Much of the information exchange between neurons is achieved with fast chemical synapses (although some types of neurons are also coupled by gap junctions). In contrast, astrocytes lack chemical synapses but are extensively coupled by gap junctions, an “astrocytic syncytium” through which various substances are transported (Scemes and Spray, 2004). Given the coupling between astrocytic calcium and modulation of synaptic transmission, and the role of synaptic communication in neuronal network dynamics, gap junction coupling between astrocytes could affect pathological neuronal dynamics. Indeed, several studies found that the levels of astrocytic gap junction protein are compromised in experimental models of epilepsy (Lee et al., 1995; Fonseca et al., 2002), but the exact implications of this aberration are not clear yet.

Experimentally, mice with deficient gap junctional coupling between astrocytes exhibit impaired potassium clearance and higher extracellular potassium accumulation during synchronized neuronal firing (Wallraff et al., 2006); thus, astrocytic gap junctions could aid in potassium buffering. These early observations were re-validated in a recent study that also revealed, in a hippocampal preparation, that gap junctions between astrocytes are important regulators of synaptic transmission (Pannasch et al., 2011). These findings suggest that astrocytic networks might control the scale of activity in neuronal networks (and perhaps vice versa) through regulation of synaptic transmission, ECS volume, and extracellular ion homeostasis.

Computational modeling of neuronal network dynamics suggests that gap junctions between neurons can either promote or suppress epileptic-like activity in a way that depends on the topology and strength of the coupling and the level of neuronal excitation. A surprising prediction of the model was that an increase in gap junctions may reduce membrane input resistance thus making a network of neurons less excitable (Volman et al., 2011a). Whether or not the same principles apply to calcium excitability in astrocytic networks is not clear. Gap junctions are usually modeled as linear electrical coupling between a pair of cells that is constant, but in fact their permeability to different ions can be modulated by several factors, including neural activity itself (Rouach et al., 2000). This adds an additional level of complexity to intercellular signaling between glial cells and implies that the “transfer function” of a gap junction could depend non-linearly on the permeating ion. One modeling study addressed the role of non-linear gap junctional transport in the context of astrocytic calcium waves (Goldberg et al., 2010) but further studies that account for their variable permeability and co-transport of different ions are needed.

Challenges associated with realistic modeling of intercellular astrocytic communication depend on the microphysiology of the cell since calcium waves propagate intracellularly at a speed of ~15 microns/s but this can vary greatly throughout the cell depending on the topology of endoplasmic reticulum in which most of the cellular calcium is stored (Yagodin et al., 1994); consequently, the cellular morphology and the exact locations of gap junctions are likely to be important determinants of intercellular communication. This issue has been addressed studying the spatial characteristics of calcium waves in networks of reconstructed astrocytes (Kang and Othmer, 2009). This study confirmed that calcium waves in astrocytes are partially regenerative; however, the model used 2-dimensional projections of reconstructed astrocytes, and whether or not the same conclusions hold in realistic 3-dimensional models remains to be shown.

Activation of astrocytic regulatory pathways, such as the release of gliotransmitters, is triggered by changes in neuronal and synaptic activity. This in turn implies that the spatial distribution of neurons and the structure of synaptic connectivity influence glial signaling. Indeed, neuronal activity can itself shape the coupling properties of astrocytic syncytium (Rouach et al., 2000). Existing network models of neuronal glial interaction, such as the ring network model (Ullah et al., 2009), have circumvented this issue by assuming simplistic network topologies and connectivity. The implications of complex neuro-glial network topologies for normal and pathological dynamics remain to be studied.

### Conflict of interest statement

The authors declare that the research was conducted in the absence of any commercial or financial relationships that could be construed as a potential conflict of interest.

## Appendix

### Modeling toolbox 1—glial mediated regulation of synaptic neurotransmitter release and neural excitability

In response to mechanical stimulation or stimulation of G-protein coupled receptors, astrocytes can release a variety of factors, the most documented ones being ATP and glutamate (Araque et al., 1999; Zhang et al., 2003). Depending on the localization of its glial release site, the released glutamate can activate receptors on postsynaptic membrane (thus modulating neural excitability) or bind to its dedicated receptors on presynaptic membrane (and thus modulate presynaptic neurotransmitter release) (see also Figure 1).

Modulation of neural excitability by glial glutamate is a relatively well understood process, as it involves activation of postsynaptic NMDA receptors (Parpura and Haydon, 2000). Thus, glial-generated NMDA current can be modeled as:

(1) $$I_{\text{ASTRO}}^{\text{NMDA}}(t)\;=\frac{g_{F}(t)-g_{S}(t)}{1+0.33\left[{\text{Mg}}^{2+}\right]\exp(-0.06V_{m})}(V_{m}-E_{\text{NMDA}})$$

(2) $$\frac{dg_{F,S}}{dt}\;=\frac{-g_{F,S}}{\tau_{F,S}}+G_{\text{ASTRO}}^{\text{GLU}}(t)$$

where *G*^GLU^_ASTRO_(*t*) represents the glutamate pulse due to glial glutamate release, the τ_*F*_ and the τ_*S*_ are the fast and slow NMDA time scales, [Mg^2+^] is the extracellular level of magnesium, and *V*_*m*_ is neuronal membrane potential. However, this detailed modeling requires knowledge of *G*^GLU^_ASTRO_(*t*) dependence on astrocytic calcium, information that is not fully available at the moment.

An alternative, phenomenological, way to model the effect of astrocytes on neuronal excitability was proposed by Nadkarni and Jung (2003):

(3) $$I_{\text{ASTRO}}=2.11\Theta \left(\log(y)\right)\log(y)$$

(4) $$y={\left[{\text{Ca}}^{2+}\right]}_{\text{ASTRO}}-196.69$$

In equations (3)–(4), Θ(*x*) is the Heaviside function (taking on value of 1 if the argument is greater than zero and value of 0 otherwise), [Ca^2+^]_ASTRO_ is the concentration of free calcium (measured in nM) in astrocyte, and current is given in pA. Fit parameters were derived from experimental data [details in Nadkarni and Jung (2003)].

Astrocytic modulation of presynaptic function is more varied. A basic assumption, supported by experimental data, is that astrocytes modulate the properties of short-term synaptic plasticity (both short-term depression and short-term facilitation). On semi-phenomenological level, short-term synaptic plasticity is well captured with Tsodyks–Markram use model which assumes that at any given time, “synaptic resource” (proportional to the number of neurotransmitter vesicles) per synaptic bouton is limited and only a fraction *x*(*t*) of it is available for release at time *t* (Tsodyks and Markram, 1997):

(5) $$\frac{dx}{dt}=\Omega_{R}(1-x)-\sum_{i}ux\delta (t-t_{i})$$

(6) $$\frac{du}{dt}=-\Omega_{F}u+U_{0}(\Gamma )\sum_{i}\left(1-u\right)\delta (t-t_{i})$$

In Equations (5)–(6), Ω_*R*_ is the rate of recovery from short-term synaptic depression, Ω_*F*_ is the rate of recovery from “astrocytic effects”, and *u* is proportional to the probability of vesicular release. The modulating effect of astrocytes on synaptic transmission is captured with the “gating function,” *U*_0_(Γ). The exact form of this gating function depends on the specifics of synapse under study [i.e., different physiological and ultra-structural parameters of a synapse will result in different functional forms of *U*_0_(Γ)]. A detailed discussion of astrocytic modulation of presynaptic function is provided in the article by De Pitta et al. in this issue, and in De Pitta et al. (2011).

### Modeling toolbox 2—glial mediated regulation of ion concentrations

In physiological conditions, potassium and chloride currents generate hyperpolarizing influences on neuronal membrane potential and thus can be considered to be seizure-suppressing; however, in pathological situations these currents become depolarizing due to the change in their reversal potentials. Reversal potentials of potassium and chloride currents are determined by the electrochemical gradients of their constituent ions:

(7) $$\begin{array}{l} E_{\text{K}}=26.64\cdot \log\left(\frac{{\left[{\text{K}}^{+}\right]}_{\text{OUT}}}{{\left[{\text{K}}^{+}\right]}_{\text{IN}}}\right)\;[\text{mV}];\\ E_{\text{Cl}}=26.64\cdot \log\left(\frac{{\left[{\text{Cl}}^{-}\right]}_{\text{IN}}}{{\left[{\text{Cl}}^{-}\right]}_{\text{OUT}}}\right)\;\left[\text{mV}\right] \end{array}$$

so the high extracellular (intracellular) concentration of potassium (chloride) shifts the reversal potential toward more positive values.

The dynamics of extracellular potassium concentration can be captured with the following equation:

(8) $$\frac{d{\left[{\text{K}}^{+}\right]}_{\text{OUT}}}{dt}=\sum I_{\text{K}}+{\left(\Delta \left[{\text{K}}^{+}\right]\right)}_{\text{DIFF}}-G$$

in which ∑*I*_*K*_ is the sum over all neuronal potassium currents (from ion channels and/or pumps), (Δ[K^+^])_DIFF_ is the contribution from potassium diffusion in extracellular space, and *G* is the term that represents buffering of extracellular potassium by astrocytes. Relatively detailed biophysical models exist to describe glial buffering of potassium, based on activation of glial potassium channels (Oyehaug et al., 2011); however, in many cases it is convenient to describe glial buffering in a semi-phenomenological way by assuming the existence of glial buffer, [B], that binds free potassium ions. Glial contribution can then be written as:

(9) $$G=\frac{-d[B]}{dt}=k_{\text{ON}}{\left[{\text{K}}^{+}\right]}_{\text{OUT}}[B]-k_{\text{OFF}}({\left[B\right]}_{T}-[B])$$

(10) $$k_{\text{ON}}=\frac{k_{\text{OFF}}}{1+\exp\left(\frac{{\left[{\text{K}}^{+}\right]}_{\text{TH}}-{\left[{\text{K}}^{+}\right]}_{\text{OUT}}}{\alpha_{\text{KG}}}\right)}$$

where *k*_ON_ and *k*_OFF_ are forward (binding) and backward (unbinding) rates, correspondingly, [B]_T_ is the total buffer concentration (usually assumed to be very high), and [K^+^]_TH_ is the threshold concentration of extracellular potassium above which glial buffer is activated (~10 mM) (Kager et al., 2000; Krishnan and Bazhenov, 2011).

Because of the electrogenic nature of Na^+^/K^+^ exchange pump bringing in 2 K^+^ ions in exchange for 3 Na^+^ ions, increase of the extracellular K^+^ (or intracellular Na^+^) concentration leads to the outward current that may hyperpolarize the cell. This current can be modeled as (Kager et al., 2000; Krishnan and Bazhenov, 2011):

(11) $$\begin{array}{cc} I^{\text{pump}}=I_{\text{K}}^{\text{pump}}+I_{\text{Na}}^{\text{pump}},I_{\text{K}}^{\text{pump}}=-2I_{\text{max}}A, & I_{\text{Na}}^{\text{pump}}=3I_{\text{max}}A \end{array}$$

(12) $$A={\left(1/(1+(K_{o\alpha }/[K_{o}]))\right)}^{2}(1/(1+(Na_{i\alpha }/[Na_{i}])))$$

where K_*o*α_ and Na_*i*α_ are baseline ionic concentrations.

The dynamics of intracellular chloride concentration can be modeled with an equation similar to equation (8):

(13) $$\frac{d{\left[{\text{Cl}}^{-}\right]}_{\text{IN}}}{dt}=\sum I_{\text{Cl}}+\frac{\left({\left[{\text{Cl}}^{-}\right]}_{\text{IN}}-{\left[{\text{Cl}}^{-}\right]}_{I\infty }\right)}{\tau_{\text{Cl}}}$$

where the characteristic time τ_Cl_ is the inverse of rate of chloride extrusion by KCC2 exporter. The KCC2 mediated effective coupling between extracellular potassium concentration and intracellular chloride concentration can be modeled as (Krishnan and Bazhenov, 2011):

(14) $$\tau_{\text{Cl}}=\tau_{\text{BASE}}+\frac{\tau_{\text{Cl}\infty }}{1+\exp\left(\frac{{\left[{\text{Cl}}^{-}\right]}_{I\infty }-{\left[{\text{K}}^{+}\right]}_{\text{OUT}}}{\alpha_{\text{KCl}}}\right)}$$

### Modeling toolbox 3—glial mediated homeostatic synaptic plasticity

Homeostatic synaptic plasticity (HSP) allows neurons and networks to adjust synaptic conductance in order to offset the effect of long-term perturbation on their firing rates (Turrigiano, 1999). Astrocytes contribute to neuronal HSP by releasing tumor necrosis factor alpha (TNFα) that binds to neuronal TNFα receptors and causes insertion and removal of AMPA and GABA receptors, correspondingly (Stellwagen and Malenka, 2006). Astrocytic release of TNFα was observed to occur following prolonged neuronal inactivity (Stellwagen and Malenka, 2006). Given that astrocytes “sense” neuronal activity through glutamate levels, it is plausible to assume that astrocytic involvement in HSP is triggered by prolonged inactivity of pyramidal (PY) neurons. In the most general form, this information can be captured with the following equations:

(15) $$g_{\text{AMPA}}=g_{\text{AMPA}}+\alpha_{\text{HSP}}^{\text{GLU}}\cdot \left(f_{0}-{\langle f\rangle }_{T,\text{PY}}\right)$$

(16) $$g_{\text{GABA}}=g_{\text{GABA}}-\alpha_{\text{HSP}}^{\text{GABA}}\cdot \left(f_{0}-{\langle f\rangle }_{T,\text{PY}}\right)$$

In Equations (15)–(16), *f*_0_ is the preset target rate of PY neurons, 〈*f*〉_*T*,PY_ is the averaged (over time and PY neurons) firing rate of PY neurons in “astrocytic domain” (Volman et al., 2011d), and α^GLU^_HSP_, α^GABA^_HSP_ are the rates of HSP convergence for specific type of synaptic receptor. Because astrocytic TNFα can only up regulate synaptic conductance, Equations (15)–(16) can only be applied in the case when 〈*f*〉_*T*,PY_ ≤ *f*_0_ (i.e., recovery from prolonged inactivity). Homeostatic down regulation of neuronal excitability is likely achieved through other neuronal mechanisms (Turrigiano, 1999). This “division of labor” between astrocytes and neurons may result in non-trivial collective activity following perturbations such as, e.g., brain trauma (Volman et al., 2011d).
